# The Role of CT Perfusion in the Evaluation and Management of Acute Ischemic Stroke—A Systematic Review

**DOI:** 10.3390/life15111693

**Published:** 2025-10-31

**Authors:** Rares C. Bobe, Roxana E. Coroiu, Adelina E. Cirstian, Camelia I. Cristescu, Diana A. Pepelea, Rosana M. Manea

**Affiliations:** 1 Clinical Laboratory of Radiology and Medical Imaging, Brasov’s Emergency County Hospital, Calea Bucuresti, Nr. 25-27, 500326 Brașov, Romaniarosana.manea@unitbv.ro (R.M.M.); 2 Faculty of medicine, Translivania University, Str. Nicolae Bălcescu, Nr. 56, 500019 Brașov, Romania

**Keywords:** CT perfusion, acute ischemic stroke, core and penumbra, collateral circulation, patient selection, prognosis, endovascular treatment, imaging validation

## Abstract

Background: CT perfusion (CTP) is increasingly used in the evaluation of acute ischemic stroke (AIS) and may complement non-contrast CT (NCCT) and CT angiography (CTA). This review aimed to assess the role of CTP in patient selection for reperfusion therapy, its prognostic value, and the influence of technical factors, collateral assessment, and post-processing software. Methods: A literature search of PubMed, DOAJ, and Google Scholar (2014–2025) identified 119 articles; after screening, 39 met inclusion criteria. Only studies on adult AIS patients investigated with CTP were included. Data were synthesized across eight thematic categories: core/penumbra estimation, prognosis, treatment selection, collateral assessment, software validation, technical parameters, reliability, and safety. Results: CTP improved identification of infarct core, penumbra, and collateral status, aiding patient selection for endovascular therapy, particularly beyond 6 h. Limitations included variability in tissue thresholds, “ghost infarct core,” and differences across software. Technical advances, such as “one-stop-shop” protocols and low-kV acquisition, reduced treatment delays and radiation. Reliability studies showed CTP to be less accurate than diffusion-weighted MRI, while safety analyses confirmed a low risk of contrast-induced nephropathy. Conclusions: CTP enhances patient stratification and outcome prediction, supporting individualized treatment strategies. Standardization of protocols and validation of software remain necessary before CTP can serve as a reliable alternative to MRI-DWI.

## 1. Introduction

Stroke represents a medical emergency, defined by a focal neurological impairment, which could be either temporary or permanent. At present, it is the third leading cause of death and acquired disability combined and the second most common cause of mortality worldwide [1]. Neurological dysfunction occurs due to a sudden decrease in the blood flow of a cerebral region secondary to a vascular occlusion (in ischemic strokes) or due to vascular rupture (in haemorrhagic stroke) causing cellular injury [2].

The primary goal in treating a patient with acute ischemic stroke is the successful restoration of cerebral blood flow (CBF) in areas with ischemia, without infarction, to reduce mortality and improve patient independence. The two interventions proven effective as reperfusion therapy are intravenous thrombolysis (IVT) and mechanical thrombectomy (MT). Alteplase remains the guideline-endorsed thrombolytic agent for acute ischemic stroke, while tenecteplase, a newer modified form with higher fibrin specificity and longer half-life, shows comparable or superior efficacy in recent trials [3]. MT is an interventional procedure that refers to a blood clot being removed from a vessel using endovascular devices and techniques such as stent retrieval, direct aspiration, or a combination of both, associated with image guidance, with proven efficacy as standard of care for patients with anterior circulation or bassilary artery occlusion since 2015 [4].

Neuroimaging is essential for evaluating patients with suspected acute ischemic stroke because it enables accurate lesion characterization. Although magnetic resonance imaging (MRI) provides higher quality images and improved delineation of affected regions, computed tomography (CT) remains the primary modality for stroke assessment. CT offers several advantages over MRI, including greater availability, improved patient tolerance, and reduced costs [5].

The visual assessment of ischemic stroke patients with the aid of CT is based on the Alberta Stroke Programme Early CT Score (ASPECTS), a 10-point score used for evaluating MCA stroke patients. ASPECTS is a 3-month outcome predictor, with a score of less than 7 points being associated with a worse outcome due to larger volume of presumed severe ischaemia [6]. Recent technological advances have increased the importance of CT angiography (CTA) and CT perfusion (CTP) in the evaluation and selection of stroke patients. These modalities may influence treatment decisions.

While CTA allows the visualization of vascular anatomy, helping in the evaluation of cerebral artery patency [7], CTP facilitates the evaluation of physiological processes by monitoring contrast enhancement with the aid of a few parameters such as cerebral blood volume (CBV), cerebral blood flow (CBF), and mean transit time (MTT), enabling the possibility of a much wider reperfusion time window [8].

A type of protocol incorporating the classical non-contrast CT (NCCT), CTA, and CTP, also known as multimodal CT imaging, has shown an improvement in acute infarction detection, evaluation of the vascular occlusion site, and the assessment of salvageable brain tissue (penumbra), with the advantage of a short acquisition time (10–15 min) [2].

As part of this multimodal approach, CT perfusion is performed by monitoring the first contrast bolus (50 mL of high-flow contrast) as it flows through the cerebral circulation, using continuous imaging over the same region of tissue (between 1 and 32 scan acquisitions are needed). Time attenuation curves are generated by placing two regions of interest (ROI), one for the arterial circulation in the contralateral middle cerebral artery (MCA) or anterior cerebral arteries (ACAs) and one for the venous flow in the superior sagittal sinus, allowing calculation of cerebral blood volume (CBV—the quantity of blood in a unit of tissue), cerebral blood flow (CBF—quantity of blood passing through a unit of tissue over a period of time), and mean transit time (MTT—mean time needed for blood to pass a certain region of brain). The interpretation of CTP maps is based on the evolution of these parameters; thus, the penumbra would exhibit reduced CBF correlated with increased MTT and normal or mildly increased CBV, while the ischemic core would present significantly reduced CBV (<2 mL/100 g) and CBF, as well as prolonged MTT. The preferred region for analysis using Perfusion CT is represented by a 4-cm slab at the basal ganglia level, encompassing representative territories of both the anterior and posterior circulations [2].

CT perfusion could also facilitate the identification of large infarcts, allowing for the differentiation between those that would still benefit from thrombolytic therapy and those that carry a significant risk of hemorrhagic transformation [8]. As Table 1 highlights, compared to NCCT, CT perfusion has several disadvantages, including a longer acquisition time, a higher radiation dose, the need for contrast administration [7], and the requirement of well-trained specialists who can effectively process and interpret the perfusion maps.

This review aims to evaluate the role of CT perfusion in the management of stroke patients, with particular emphasis on the significance of CTP parameters for patient selection in reperfusion therapies and their prognostic value.

The assessment of CTP’s significance in widening the reperfusion time window (6–24 h) is one of the secondary focuses of the current paper.

Additional objectives of this article include comparing different CTP postprocessing software packages and their impact on infarct core volume estimation, as well as identifying technical factors influencing CTP results, such as interobserver variability, acquisition parameters, and radiation dose. A secondary objective of the current paper is to evaluate the predictive value of CTP parameters on collateral status, infarct progression, potential hemorrhagic transformation risk, and 90-day functional outcome.

Assessment of safety issues related to contrast media administration and radiation exposure represents one of the side points of this paper.

The impact of standardized and integrated protocols (e.g., multimodal CT) on workflow efficiency and time to treatment, both after the onset of symptoms and upon arrival in the ED, was also analyzed.

## 2. Materials and Methods

**Figure 1:**
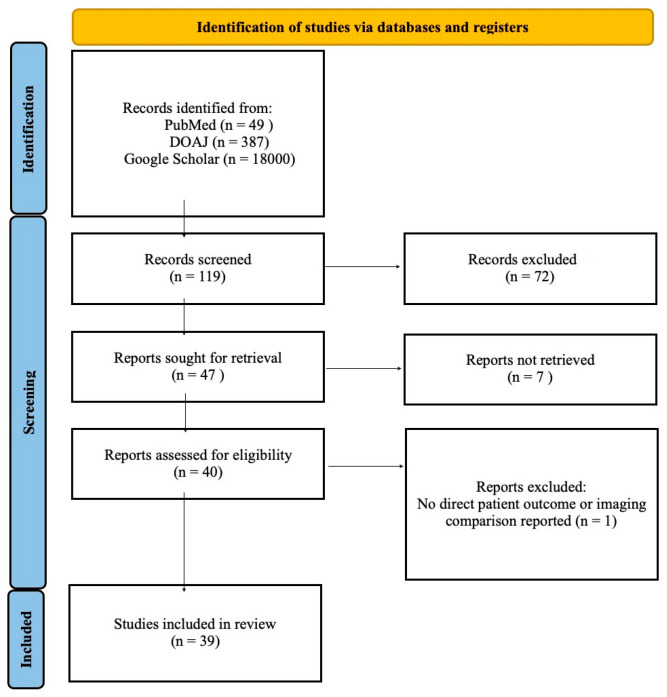
PRISMA flowchart of current literature review-The PRISMA flowchart shows the selection process of the included studies.

This literature review employed a semisystematic approach, involving a search of international databases including PubMed, DOAJ, and Google Scholar as shown in the PRISMA flowchart in Figure 1. Search terms included “CT perfusion,” “acute ischemic stroke,” and “perfusion imaging in stroke.” Non-English language articles were excluded.

During the initial search of databases, we found 49 results on PubMed, 387 on DOAJ, and over 18,000 in Google Scholar. Of these, 119 were initially evaluated by title and abstract, and 72 were excluded due to ineligibility. Of the 47 that remained, only 40 were open-access; one was excluded because its primary focus was not on clinical outcomes but on machine learning. We included articles published in the last eleven years (2014–2025) in our paper.

Studies that met the inclusion criteria were those with: (1) adult human subjects in which the (2) diagnosis (confirmed or suspected) was acute ischemic stroke (AIS) (3) articles that investigated subjects by CTP besides other methods, such as MRI, NCCT, and multimodal CT, and (4) even studies that had compared different CTP processing software for validation in order to come up with reliable alternatives for a faster workflow and reduced time to treatment.

Articles that were designed as systematic reviews or meta-analyses were not included in this paper in order to avoid redundancy and to maintain focus on original studies.

After analyzing the full text versions of the found articles, the eligible ones were extracted and grouped into a general table, which represented the main structure for this review.

The studies included have been grouped by their main objective in eight thematic categories: core/penumbra estimation, software comparison/validation, treatment selection, technical/imaging parameters, collateral circulation, prognosis, safety, and reliability, and the main results were compared in a descriptive manner, as this paper doesn’t represent a statistical analysis.

## 3. Results

A total of 39 articles published between 2014 and 2025 that met the inclusion criteria were analyzed. The included studies involved patients diagnosed with acute ischemic stroke who underwent CT perfusion, often in combination with non-contrast CT (NCCT), CTA, or MRI. For clarity, the articles were categorized into eight thematic groups: (1) core/penumbra volume estimation, (2) treatment selection, (3) prognosis and functional outcome prediction, (4) collateral circulation grade estimation, (5) software comparison/validation, (6) technical and imaging parameters, (7) reliability, and (8) safety.

**Figure 2:**
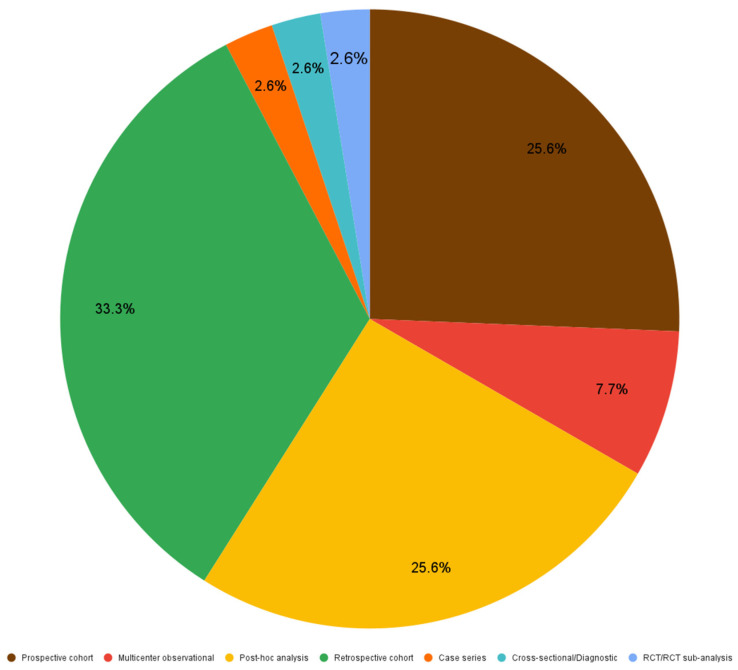
Articles distribution based on study design-The chart highlights the proportion of retrospective, prospective, post-hoc, multicenter, RCT, cross-sectional and case-series studies.

Figure 2 demonstrates that out of 39 included articles, 33.3% (13 articles) were designed as retrospective studies, 25.6% (10) were prospective studies as much as post-hoc analyses, 7.7% (3) were multicenter observational studies, while RCT/RCT sub-analyses, cross-sectional studies and case series represented 2.6% (1) each.

**Figure 3:**
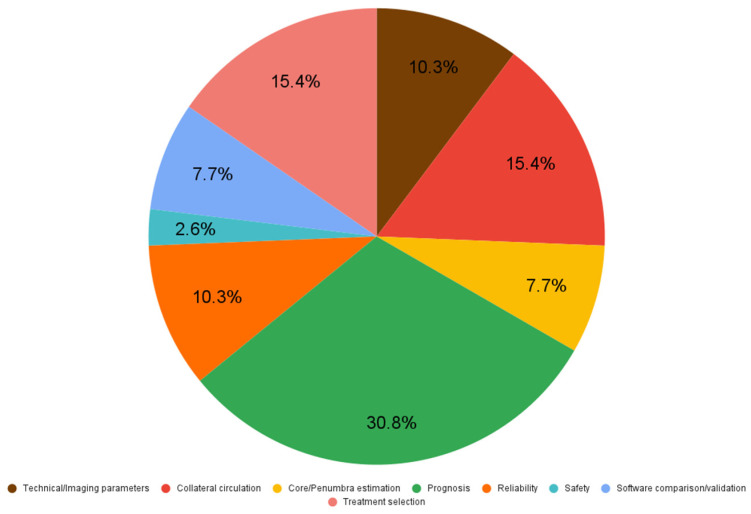
Articles distribution based on thematic categories-The chart illustrates the proportion of studies focusing on prognosis, treatment selection, collateral assessment, technical parameters, reliability, core/penumbra estimation, software validation and safety.

Based on the thematic category of their main objective, as shown in Figure 3, out of all included papers: 30.8% (12 articles) had the main focus on implications of CTP in prognosis of stroke in patients investigated with this method; the importance of CTP in treatment selection, as well as collateral grade estimation accounted for 15.4% (6) each; studies investigating the impact of different protocol changes on the technical or imaging parameters also the reliability of different methods were as much as 10.3% (4) each; papers that had the main focus on core/penumbra volume estimation and software comparison/validation represented about 7.7% (3) each; studies concentrated on the safety aspects represented only 2.6% (1).

The minimum number of patients included in the studied articles was n = 15 [10], and the maximum number was n = 4249 [11], with a mean of 330.74 patients and a median of 144 patients.

### 3.1. Core/Penumbra Estimation

All the studies included in this category have assessed the accuracy of CT perfusion in quantifying the infarct core and penumbra volume compared to other methods, while observing various CTP limitations.

A retrospective study that investigated n = 59 patients demonstrated that CTP estimation of core and penumbra volume has better specificity than traditional visual scoring systems like ASPECTS (Alberta Stroke Programme Early CT Score-conventional or automated) in case of large infarcts, and the general accuracy between the analyzed methods was similar [12].

Another issue raised by a retrospective study on n = 43 subjects with AIS, investigated with CTP within 6 h of symptom onset, is the thresholds for the ischemic core and penumbra, especially between gray matter (GM) and white matter (WM). It was shown that the usage of unified thresholds may lead to an overestimation of lesions in the WM and to an underestimation of the lesions in GM, setting the need for separate thresholds between the two [13].

In a retrospective analysis of n = 123 patients observed that the subjects who were rapidly referred to reperfusion therapy had a tendency to overestimate the infarct core at baseline CTP compared to the follow-up study, with a difference greater than 10 mL, a phenomenon termed “ghost infarct core” [14].

### 3.2. CT Perfusion Implications in Prognosis of Outcome

The primary objective of the articles included in this section was to examine the potential implications of CT perfusion parameters on outcome prediction in patients diagnosed with acute ischemic stroke.

A multinational team investigated the associations between various metabolic factors, including baseline blood glucose and hemoglobin levels, and the accuracy of infarct core estimation in a sample of n = 162 patients retrospectively analyzed. They demonstrated that in anemic and/or hyperglycemic AIS patients, the volume of the infarct core can be overestimated, and metabolic factors should be considered when interpreting CTP images of these patients [15].

A post-hoc analysis of MRCLEAN [16] and CRISP [17] studies, which investigated n = 228 patients (n = 127 from MRCLEAN, n = 101 from CRISP), has correlated the basic CTP parameters, such as cerebral blood volume (CBV), cerebral blood flow, and mean transit time (MTT) with the final size of the infarct, allowing identification of lesion expansion dynamics [18]. A similar finding was supported by another post-hoc study, which analyzed n = 151 subjects and stated that hypoperfusion volume and infarct core volume could predict the size of the final infarct, with the combination providing superior estimator accuracy [19].

This retrospective study [20] on n = 306 people with AIS, with early neurological deterioration of more than 2 points NIHSS score, concluded that hypoperfusion in the lenticulo-striate territory was associated with early neurological deterioration after thrombolysis [20]. A multinational post-hoc analysis that investigated n = 115 AIS patients revealed a correlation between hypoperfusion in the white matter of the contralateral hemisphere and the progression of small vessel disease (SVD) [21].

In a case series of n = 15 patients diagnosed with chronic stenosis (unilateral internal carotid artery -ICA stenosis; MCA stenosis/occlusion), the values of MTT correlated with a higher occurrence rate of ischemic stroke in cases with MTT values greater than 6 s [10].

Another study that retrospectively analyzed n = 408 AIS patients who underwent baseline CTP, follow-up imaging, and received mechanical thrombectomy (MT) showed associations between certain parameters (rCBF < 30%, Tmax  >  6 s, and CBV  < 38%) and the risk of hemorrhagic transformation [22].

In patients with medium vessel occlusions, treated via MT, CT perfusion showed that dimensions of core and penumbra were associated with infarct size and outcome, as a retrospective study of n = 66 subjects found out, concluding that a smaller core and penumbra on the baseline perfusion CT correlates with a better outcome at 90 days post MT [23].

With the aid of CT perfusion, another study prospectively analyzed n = 144 patients with symptomatic intracranial atherosclerosis (sICA) and observed that different infarct patterns are associated with the severity of hypoperfusion, leading to more challenging neurological recovery in the short term [24].

In a large prospective cohort investigation, after the inclusion of n = 1374 patients with suspected AIS within 9 h from onset that were investigated via NCCT, CTA, and CTP, studied the association between CTA and CTP baseline parameters and the 90-day outcome (quantified by modified Rankin-Scale mRS), proving a decent degree of predictability for clinical outcome, but limited additional value to NCCT and clinical data in an unselected population [25].

In a population of n = 156 patients with acute ischemic stroke who underwent successful reperfusion, a post-hoc analysis found a better association between CTP’s core volume and clinical outcome, exceeding the visual evaluation provided by the ASPECTS [26].

Another retrospective analysis of n = 118 patients with intracranial atherosclerosis (ICAS), utilizing CTP, computational fluid dynamics (CFD), and machine learning, has demonstrated an enhanced ability to differentiate stroke mechanisms, enabling the development of personalized treatment strategies [27].

### 3.3. Treatment Selection

Perfusion CT may play a crucial role in determining the most suitable treatment strategy for patients.

In a post-hoc analysis of the IMS-III trial, on n = 648 patients divided into 3 groups: investigated by NCCT, CTA, and CTA + CTP, it was demonstrated that CTA either alone or associated with CTP could offer shorter time from patient’s arrival in the ED to treatment compared with NCCT, although CTP itself did not prove superiority in time to treatment reduction; CTA was also associated with better outcomes of patients selected for MT [28].

On the other hand, the sub-analysis of the randomized clinical trial PRACTISE, n = 271 patients, divided into two groups, investigated either with NCCT or via multimodal protocol, found a decrease in the amount of people selected for IV thrombolysis, due to additional information, but with no significant difference in time-to-treatment or in the 90-day outcome, between the two groups [29].

A large multicenter observational study evaluating n = 4249 subjects has concluded that perfusion CT enables a more accurate selection of patients for MT, both in the early (0–6 h) and late (6–24 h) time windows, resulting in a decrease in the rate of futile recanalization and mortality [11].

CRISP has shown in a prospective study on a cohort of n = 190 people, that the patients who presented “target mismatch” on CTP would benefit from MT up to 18 h from symptom onset, supporting the utility of perfusion in selecting patients in late window (>6 h) for treatment [17].

Similarly, a prospective study with n = 107 subjects, has shown the superiority of CTP in the selection of patients with large infarcts, based on significant “target mismatch” presence, showing an increased hemorrhagic transformation risk in its absence [30].

A prospective study that investigated the association between imaging profiles and functional outcome in n = 361 people, has shown that in patients with favorable profile on both NCCT and CT, the functional independence rate after MT was higher than in patients with discordant profiles, showing that favorable NCCT in patients with unfavorable CTP, which was associated with increased rates of sICH and mortality [31].

### 3.4. Collateral Assessment

Collateral assessment of an acute ischemic stroke patient could represent an important step regarding both treatment decisions and outcome prediction.

Two of the included studies have been investigating the association between collateral circulation, assessed by single-phase CTA (sCTA) and whole-brain CTP/dynamic CTA (dCTA), with the 3-month outcome. One of these articles has retrospectively evaluated n = 70 patients with proximal AIS within 9 h from onset, evaluating arterial collateral circulation [32] and the other has investigated the extent and velocity of cerebral venous flow (CVF) in n = 88 patients with proximal AIS within 9 h from onset too [33]; The results have shown a better ability of dynamic CTA in the assessment of collateral circulation [32] and CVF compared with single-phase CTA [33] and a better correlation with the 90-day outcome [32,33].

The post-hoc analysis of the SWIFT-PRIME trial [34], which evaluated n = 158 patients with baseline CTP imaging, found an association between relative cerebral blood volume (rCBV), hypoperfusion index ratio (HIR), and infarct size, with rCBV representing a predictor of infarct growth after reperfusion therapy [35].

A retrospective study which evaluated maximum cerebral blood flow of collateral vessels (cCBFmax) using CT perfusion in a cohort of n = 296 patients, demonstrated a correlation with CTA collateral grading and the ability of cCBFmax to discriminate between mechanisms of stroke, including large arterial atherosclerosis (LAA) and cardioembolic events [36].

In a prospective study on a population of n = 178 patients with acute ischemic stroke investigated with CT perfusion and dCTA, a parameter based on delay time (DT) (DT > 6 s for good collateral status areas/DT > 2 s for ischemic areas) has successfully quantified collateral status, comparable with dCTA, showing prediction value for the infarct size and outcome [37].

In a post-hoc analysis of DEFUSE-3 trial [38], after the analysis of n = 123 AIS patients, a link between collateral status and 24-h predicted infarct size was described, the patients with poor collaterals were the ones that had shown infarcts greater than predicted on the 24 h follow-up imaging, while good collateral status limited the evolution of infarcted tissue [39].

### 3.5. Software Comparison/Validation

The increasing use of CT perfusion for managing patients with acute ischemic stroke has led to the development of several software packages designed to help specialists interpret perfusion maps and streamline workflow time. As each package uses different algorithms and thresholds, this may influence the accuracy and comparability of ischemic core and penumbra quantification. Therefore, studies in this category have focused on comparing their performance, as well as testing and validating novel methods.

Koopman et al. conducted a post-hoc analysis of n = 35 patients with AIS who underwent baseline CTP and had their images post-processed using three software programs: RAPID, SYNGO.VIA, and INTELLISPACE PORTAL (ISP). The results showed that the estimation of the ischemic core had significant variance. The best concordance with the RAPID software (validated) was using Syngo.via with an additional smoothing filter; ISP has frequently overestimated core size. RAPID has shown the best prediction value regarding final infarct size [40].

A retrospective study that compared CTP images of n = 159 patients, processed both with RAPID and U-GUARD, has shown good agreement between the two software packages in outcome prediction after MT, with RAPID being more specific for penumbra volume, while U-GUARD being slightly more specific for core volume [41].

A multicenter observational study that investigated n = 115 AIS patients using CTP and vascular territory mapping (VTM) techniques failed to demonstrate a correlation between VTM, visual collateral assessment, and 90-day clinical outcome [42].

### 3.6. Technical/Imaging Parameters

The articles included in this section focus on a few technical aspects and image quality of different protocols used in perfusion CT.

Sanossian et al. in a multicentric observational study with n = 1700 patients have concluded that NCCT remains the main choice in cases of acute ischemic stroke, nevertheless the use of multimodal approach has increased from 4% (2005–2006) to 26% (2011–2012) being associated with a faster referral of the patient to reperfusion therapy [43].

A prospective study on a n = 100 AIS patients’ cohort has evaluated the “one-stop-shop” protocol, which promoted the addition of CTA and CTP to the conventional CTP, and observed a decrease in examination time (of approximately 28%), radiation exposure, and contrast dose, streamlining the workflow in the emergency department [44].

A prospective study on n = 150 stroke patients, which investigated the use of a 70 kV protocol in acquiring CTP images, resulted in a reduction in radiation exposure of approximately 37%, with image quality similar to that of the traditional 80 kV protocol [45].

In a cohort of n = 88 patients with suspected posterior circulation ischemic stroke, CT perfusion increased the sensitivity for detection from 31–33% to 74% (especially in cases of cerebellar lesions) when added to NCCT + CTA, resulting in a higher negative predictive value [46].

### 3.7. Reliability

The reliability of CT perfusion imaging in terms of diagnostic accuracy and clinical utility, particularly in stroke patients, may be influenced by several factors, including interobserver variability and technical issues, as well as comparisons with established methods such as diffusion-weighted imaging (DWI). The focus of the studies in this category was to assess these aforementioned factors.

El-Tawil et al. in a study involving n = 24 perfusion imaging series assessed by 57 independent observers, demonstrated good to excellent interobserver agreement regarding MTT, DT, and penumbra, with a low decrease in agreement regarding CBV and CBF. It also concluded that the level of expertise and extended coverage of the scan had a positive influence on the study’s results [47].

In a prospective study of n = 26 patients with carotid artery stenosis subjected to carotid artery stenting (CAS), the “false ischemic penumbra” phenomenon was identified in 38.5%. After revascularization therapy, CBF, MTT, TTP, and Tmax have normalized, confirming that no true ischemic areas were involved. This suggests that severe carotid stenosis may lead to errors in the interpretation of CTP [48].

A retrospective study by Caefer of n = 55 patients with anterior vessel occlusion showed a correlation between CT perfusion and DWI, but with significant errors, as CT-CBV underestimates ischemic volumes. Authors have concluded that CT perfusion is useful for analyzing groups rather than individual triage of stroke patients [49].

A retrospective study of n = 58 patients has demonstrated significant variability in CBF maps derived from CTP, with a tendency to overestimate the ischemic core volume compared to DWI, without any single threshold being able to substitute for the accuracy of MRI-DWI [50].

### 3.8. Safety

A post-hoc analysis of n = 182 patients from the DEFUSE-3 trial [38] evaluated the safety of contrast media administration in case of multimodal approach of stroke patients prior to MT. The authors observed a low decrease in serum creatinine levels within the first 24 h after treatment, with no significant difference between those treated via MT and those receiving best medical therapy only. The incidence of contrast-associated renal impairment was low, with no patient needing dialysis [51].

## 4. Discussion

This systematic review synthesized data from 39 studies published between 2014 and 2025, evaluating the role of CT perfusion (CTP) in the assessment and management of acute ischemic stroke. The results demonstrate that CTP provides valuable information for estimating infarct core and penumbra volumes, assessing collateral status, and guiding patient selection for reperfusion therapy, particularly in extended time windows beyond six hours. CTP-derived parameters such as cerebral blood volume (CBV), cerebral blood flow (CBF), and mean transit time (MTT) showed good correlation with clinical outcome and hemorrhagic transformation risk, although their accuracy is affected by threshold variability, technical factors, and differences among post-processing software packages. The analysis also revealed that CTP improves workflow efficiency when incorporated into multimodal CT protocols, while maintaining a low risk of contrast-induced nephropathy. However, studies comparing CTP with diffusion-weighted imaging (DWI) consistently reported limited reliability of CTP for precise core estimation, reinforcing the need for multimodal imaging and standardized acquisition protocols. Overall, CTP represents a powerful but still evolving tool that complements, rather than replaces, established MRI techniques in the comprehensive evaluation of stroke patients.

### 4.1. Core/Penumbra Estimation

Although CT perfusion can provide valuable information about core and penumbra volume estimation, especially when compared to visual scoring systems [12], the analyzed studies also suggest significant variability in its performance [13,14], influenced by reperfusion therapy or ischemia thresholds [14].

### 4.2. CT Perfusion Implications in Prognosis of Outcome

Available data support the value of CTP in predicting acute ischemic stroke clinical outcome, but it could also be influenced by a variety of factors. It has been demonstrated that metabolic variables, such as hemoglobin and blood glucose levels, can affect the accuracy of CT perfusion in estimating ischemic core volumes [15]. Several post-hoc analyses have shown a correlation between hypoperfusion, ischemic core volumes, and the final size of the infarction, with the ability to predict the lesion expansion dynamics [18,19]. Other studies have linked hypoperfusion in specific territories (lenticulo-straite region, whitematter) with early neurological deterioration [20] and the progression of SVD [20]. Perfusion CT was also associated with 90-day functional outcome [23,26] and with the risk of hemorrhagic transformation after MT [22]. Although some studies have shown limited predictive value of CTP parameters compared to NCCT and clinical data [25], other investigations suggested that the combination of CT perfusion with computational fluid dynamics (CFD) and machine learning could facilitate stroke mechanisms clarification, allowing individualized treatment strategies [27], paving the way for new research directions in the use of CTP.

### 4.3. Treatment Selection

Although the analyzed studies have shown no significant improvement in treatment times in patients selected with CTP, compared to NCCT [28]. CTA and CTP could provide additional information, which could aid in selecting patients who would benefit from MT [28,29], thereby reducing the risk of futile recanalization or mortality [11]. The presence of target mismatch on CTP could aid in selecting subjects who would benefit from MT in the late window [17,30]. Additionally, imaging profile discordance (between NCCT and CTP) could help identify patients with an increased risk of sICH and mortality [31].

In summary, although CT perfusion does not significantly reduce time to treatment, it provides valuable prognostic and selection information. This modality may help identify patients most likely to benefit from endovascular treatment, particularly in extended time windows.

### 4.4. Collateral Assessment

Available data show an association between collateral status and prediction of clinical outcome in ischemic stroke patients [39]. CTP parameters, such as rCBV, cCBFmax, and DT index, may quantify collateral status with accuracy similar to dCTA, showing superiority over qualitative measures and a good predictive value for clinical outcome [35,36,37]. Additionally, collateral assessment could help improve the stroke etiology identification and explain the discordance between predicted and actual infarct size [36], allowing a better patient stratification and selection for therapeutic strategies.

Overall, collateral assessment through perfusion CT and advanced CTA methods emerges as a promising prognostic tool, which, in conjunction with clinical data, can aid in selecting and supporting tailored reperfusion strategies.

### 4.5. Software Comparison/Validation

While RAPID remains the most stable and reliable reference when it comes to CT perfusion post-processing software packages, there are new solutions which show promising results in estimation of core and penumbra volume such as Syngo.via (with minor adjustments) [40] and U-GUARD, although small differences in specificity emphasize the need for further validation [41]. VTM has not yet shown reliable evidence in its ability to assess collateral status or predict outcomes [42]. The results of these studies underscore the need for standardization and crossvalidation of software packages to ensure accurate patient selection in clinical practice.

### 4.6. Technical/Imaging Parameters

The analysis of the articles included in this review suggests an increasing trend in the use of CT perfusion as part of a multimodal approach in stroke patients [43].

Other findings confirm that the use of new protocols as “one-stop-shop” and tension reduction in CT tube from 80 kV to 70 Kv allows a reduction in examination time, a decrease in radiation exposure and contrast dose while maintaining the same image quality and diagnostic accuracy [44,45].

Perfusion imaging also demonstrates its utility in diagnosing strokes in the posterior circulation territory [46].

Nevertheless, it must be acknowledged that diffusion-weighted imaging (DWI), although highly sensitive for detecting acute ischemia, also presents certain limitations. DWI lesion volume does not always correlate precisely with the final infarct size, particularly in the early hours after symptom onset, and may overestimate or underestimate the infarcted tissue depending on reperfusion dynamics [47,48,49,50]. Late follow-up imaging using non-contrast CT or T2-weighted MRI remains the gold standard for defining the definitive infarct. Furthermore, DWI reflects restricted diffusion rather than metabolic viability and therefore does not delineate the ischemic penumbra. Infarct core can also be estimated using very low cerebral blood flow (CBF) values on CT or MR perfusion, while penumbral tissue is typically characterized by a mismatch between CBV and CBF parameters rather than by oxygen extraction fraction (OEF), which would be the true physiological gold standard [22,47,48]. Consequently, both DWI and CTP should be interpreted in the context of clinical evolution and multimodal imaging follow-up, acknowledging that none of these techniques fully represent the dynamic pathophysiology of ischemic injury.

### 4.7. Reliability

Regarding the reliability of CT perfusion imaging, available data has shown good to excellent interobserver agreement, especially regarding time parameters and penumbra maps [47], with higher variability in the case of CBF and CBV, which highlights the need for standardization and specialization. Anatomical and pathological factors such as carotid artery stenosis could also influence the interpretation of CTP imaging, potentially causing the “false ischemic penumbra” phenomenon [48].

When compared to validated methods, such as DWI, CT perfusion has been shown to have major limitations, with the potential for overestimating the ischemic penumbra and underestimating the ischemic core [49,50]. With that being said, perfusion imaging has been shown valuable for research [50] and patient selection for treatment; however, its limited reliability justifies permanent correlation with clinical data and the use of superior imaging methods, such as DWI.

### 4.8. Safety

Although the included article suggests that utilization of CT perfusion as well as DWI for acute ischemic stroke patient selection followed by MT doesn’t associate with significant risk of renal impairment [51], there is need for more dedicated studies to confirm this hypothesis, as the cited article was a post hoc analysis and the monitorization was limited only to 24 h.

### 4.9. Limitations

The current review followed a light systematic approach, including only open access studies, with the exclusion of meta-analyses and other systematic reviews; therefore, it may have resulted in some relevant data omission. The nature of the descriptive comparison between the articles’ results did not allow for statistical analysis.

## 5. Conclusions

The evaluation and integration of the results from the analyzed articles demonstrate that CT perfusion represents a valuable asset in the management of patients with acute ischemic stroke and outcome prediction, playing an important role in stratifying patients based on the stroke mechanism and facilitating the selection of patients who would most benefit from reperfusion therapy, allowing personalized treatment strategies, with the help of currently developing tools as collateral assessment, machine learning or computational fluid dynamics. The variability in thresholds for ischemic core or penumbra, as well as the variability between different software packages, emphasizes once again the need for a more standardized approach, as CTP still cannot be considered a reliable replacement for DWI in clinical practice.

Further multicentric studies and uniformization of acquisition protocols are needed in order to integrate CTP into daily practice.

## Figures and Tables

**Table 1:** Role of each CT method (NCCT, CTA, and CTP) and comparison of radiation dose (expressed as Dose-Length Product, DLP-mGyxcm) in acute ischemic stroke imaging [2,9].

	**NCCT**	**CTA**	**CT Perfusion**
**Role**	Presence/absence of hemorrhage Infarcted tissue area	Vascular anatomy of possible occlusion site Collateral status	Physiopathological processes CBV, CBF, MTT Infarct core penumbra
**Radiation dose (DLPmGyxcm)**	754	1441	1424

## Data Availability

All data used in this systematic review are from previously published studies, which are cited and available within the article and its references. No new data were generated.
